# Cardiac Tamponade as a Life-Threatening Complication of Laparoscopic Antireflux Surgery: The Real Incidence and 3D Anatomy of a Heart Injury by Helical Tacks

**DOI:** 10.1089/lap.2017.0713

**Published:** 2018-09-11

**Authors:** Mehmet Ali Yerdel, Ozan Şen, Utku Zor, Simay Kara, Bülent Acunaş

**Affiliations:** ^1^ İstanbul Bariatrics and Advanced Laparoscopy Center, Istanbul, Turkey.; ^2^ Department of Cardiology, Acıbadem Fulya Hospital, Istanbul, Turkey.; ^3^ Department of Radiology, Acıbadem University Medical School, Istanbul, Turkey.; ^4^ Department of Radiology, İstanbul University Medical School, Istanbul, Turkey.

**Keywords:** cardiac tamponade, laparoscopic antireflux surgery, helical tack, complication

## Abstract

***Background:*** Cardiac tamponade (CT) is a dreadful complication of laparoscopic antireflux surgery (LARS) with unknown incidence, and preventive measures are yet to be defined. Incidence during LARS with respect to usage/configuration of graft deployment is analyzed. Three-dimensional (3D) analysis of tack distribution provided anatomical insight to prevent cardiac injury.

***Materials and Methods:*** Data regarding the usage and configuration of graft deployment are retrieved from the prospective database. Grafting was “posterior” or “posterior + anterior.” Incidence of CT in all hiatoplasties is calculated. Tomography is reconstructed in 3D, showing the spatial distribution of the tacks. Tacks are numbered in the surgical video. Corresponding numbering is applied to the tacks in any particular tomography slice, utilizing the 3D images as an interface. A numbering-blinded radiologist is asked to identify the offending and the nonoffending tacks as the cause of tamponade. Tack-to-pericardium distances are recorded. Tacks having no measurable distance from the pericardium are regarded as offensive.

***Results:*** One CT occurred in 1302 consecutive LARS (0.076%). The incidence is 0% when “no” (379) or “posterior” (880) graft is used as opposed to 2.3% rate in “posterior + anterior” (43) grafting. The distribution of “offensive,” “nonoffensive but nearest,” and “safe” tacks followed a pattern. All offensive tacks belonged to the anterior graft fixation, which we referred as the critical zone.

***Conclusion:*** CT during LARS is rare, and associated with graft fixation anterior to the hiatal opening. Avoiding graft fixation to the critical zone may prevent cardiac injury.

## Introduction

Iatrogenic cardiac tamponade (CT) is a life-threatening complication of laparoscopic antireflux surgery (LARS).^1–11^ The real incidence during LARS is unknown, and there is reason to believe that it is an “under-reported” complication.^8^ No previous report has provided scientific anatomical and/or surgical landmarks to avoid CT.

As a center specifically involved in LARS and using graft fixation selectively,^12,13^ the incidence of CT with respect to the “usage” and “configuration” of graft deployment in 1302 consecutive patients is presented. In addition, using the three-dimensional (3D) ability of tomography in conjunction with the surgical video, the exact distribution of all 16 titanium tacks with special reference to their measured proximity to critical structures (pericardium, aorta, and vena cava) allowed detailed anatomical insight into a tack fixation-related heart injury. Findings of the incidence and anatomical study have practical surgical implications that may be useful to prevent iatrogenic CT.

## Materials and Methods

### Incidence

**FIG. 1.**
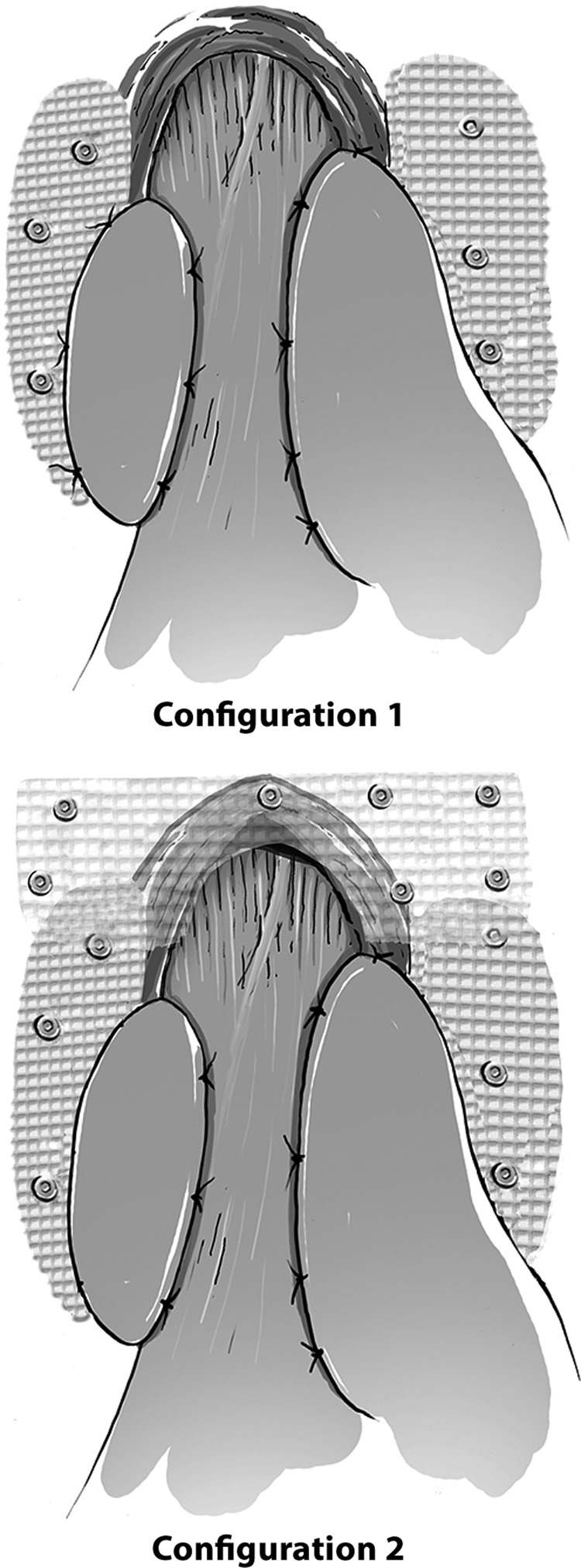
Configurations of graft deployment.

All operations were done by the principal author (M.A.Y.). Our standard approach in varying degrees of surgical severity has been reported previously.^12–14^ Currently, we abandoned doing Nissen's in favor of Toupet's after our results demonstrated no antireflux benefit in contrast to a significant increase in complications with Nissen's.^14^ After the hiatal opening was partially closed by sutures, grafts were fixed in two distinct configurations by tacks, depending on the size of the defect. In all mesh augmentations, a U-shaped graft was cut and positioned through the back of the esophagus (configuration 1 in Fig. 1). If the anterior hiatal opening is still defective after the fundoplication was completed, a second additional rectangular graft was cut and fixed right anterior to the hiatal opening (configuration 2 in Fig. 1).

All grafts used are monofilament polypropylene (Prolene™; Ethicon, Somerville, NJ) and all fixations are done by helical tackers (ProTack™; Covidien, Mansfield, MA), without exception.

Data regarding graft usage and the configuration of the deployment were retrieved from our prospective database. Incidence of CT in all configurations was calculated.

### Surgical anatomy

#### Case report

**FIG. 2.**
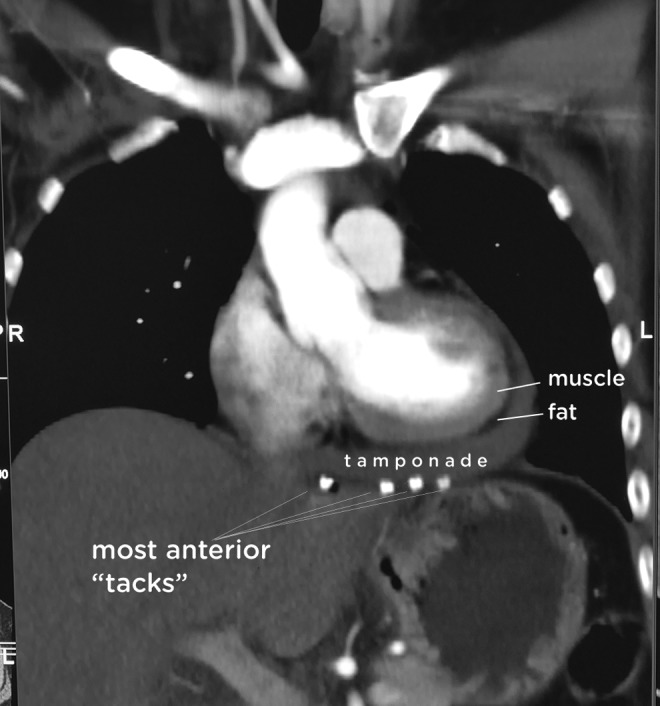
Contrast tomography showing the tamponade and the offending helical tacks.

A 42-year-old woman was referred to our center with severe gastroesophageal reflux disease (GERD) 5 years after having an open Nissen's procedure without mesh augmentation elsewhere. She had a giant recurrent hernia and pH-meter proven reflux; manometry showed moderate dysmotility. Surgery took 3 hours because of adhesions, and a configuration 2 graft deployment was performed. Recovery was uneventful until the 6th postoperative hour when orthostatic hypotension (systolic 85–90 mmHg) with an increased heart rate (120–125/min), unresponsive to fluid challenge, became evident. There was no chest pain, her enzyme levels and electrocardiography were normal. As hypotension in resting supine position and shortness of breath became evident, a bedside echocardiography and tomography were performed. Both revealed CT, and tomography also identified several “tacks” as the cause (Fig. 2). She was immediately transferred to cardiac intensive care for a possible intervention to release the tamponade. Heart rate and blood pressure slowly improved with titrated hydration under monitoring. She was followed up by serial echocardiograms, which showed slight decrease in tamponade. On the 4th day, she was transferred to the ward with a 90-minute heart rate and 115–120 mmHg systolic blood pressure. She was discharged from the hospital on the 10th postoperative day. A year after surgery, she was in perfect health, without any need for antiacid medications. Control imaging a month and a year after surgery confirmed the absence of any fluid collection.

The hyperdense nature of the element titanium in tomographic imaging allowed the anatomical analysis in this report. Contrast tomography was reconstructed in 3D with special intent to show the spatial distribution of all 16 titanium tacks, which were circumferentially deployed all around the hiatal opening (Supplementary Video S1; Supplementary Data are available online at www.liebertpub.com/lap). The video of the operation was reviewed, and starting from the first fixation, all tacks were numbered consecutively (Supplementary Video S2). Supplementary Video S2 also shows the application of surgical numbering to the corresponding tacks in the 3D image. The axial, sagittal, and coronal sections were re-evaluated in-depth, with the guidance of the 3D restoration (Supplementary Video S3). Targeted marking of any particular tack, by simultaneously identifying the same tack in all sections, allowed us to number all the tacks appearing in any particular tomography slice, according to the surgical numbering. A radiology expert, blinded to the numbering, was asked to assess the tomography slices to verify the positioning of the 16 tacks as probable causes of cardiac injury (coauthor B.A.). The closest distance from each tack to the pericardium was measured (Supplementary Video S4). Tacks without any measurable distance from the pericardium were evaluated as “offensive” as the probable cause of tamponade, whereas tacks with a clear measurable distance were regarded as “nonoffensive” by the radiologist. The number of all tacks were then uncovered.

Furthermore, tack distribution with special reference to their proximity to the aorta and vena cava was measured.

## Results

### Incidence

Between March 2004 and April 2017, 1302 consecutive LARS procedures were performed. There was no mortality and conversion. Among 1302 LARS, 379 (29%) repairs were done without graft augmentation, 880 (67.7%) had configuration 1 graft deployment, and 43 (3.3%) had configuration 2 graft deployment. The only tamponade occurred in the reported case, and there is strong evidence (Fig. 2; Supplementary Video S5) that it is related to the fixation of the anteriorly placed second graft.

The incidence of CT in entire series was 0.076%, and it was 0.108% in 923 mesh-augmented hiatoplasties. The incidence was 0% when no graft (379) or only posterior mesh augmentation (880) was used. In 43 patients with anterior graft fixation, the incidence was 2.3% (1/43).

### Surgical anatomy

Supplementary Video S4 shows the coronal and axial sequential tomographic slicing and presents all the tacks and the measurements. The radiologist could not define any measurable distance between the tack and the pericardium in tacks numbered 10, 11, 13, 14, and 15 in all tomography slices. Tack numbered 16 was also regarded as offensive as there was no measurable distance from the pericardium, at least in several slices. All of these six tacks were reported as having the potential of being the cause of cardiac injury.

None of the other 10 tacks were found to be offensive as there was a clear measurable distance between the tack and the pericardium in all slices. The closest tacks to the heart were tack numbers 7, 12, and 1, and the shortest measured distances from them to the pericardium were 0.59, 0.79, and 0.86 cm, respectively. These were the nearest nonoffenders. The rest seven tacks, radiating inferiorly away from the heart, were anatomically irrelevant as far as the cause of tamponade was concerned.

Among the six offenders, tack numbers 10, 11, 13, and 14 actually form a horizontal line, underlining the base of the heart from right to left (Fig. 2). These four were also the most anteriorly applied tacks, depicting a line just in front of the hiatal opening. Other two offenders, tacks numbered 15 and 16, were adjacent to this line, and also anteriorly located, but 1 cm posterior to the anterior tack line, and at the left side of the hiatus (Supplementary Video S5).

In fact, the offender description relates to a particular area, corresponding to the zone covered by the anterior rectangular strip graft as demonstrated and referred as the “critical zone.”

Among nonoffending tacks, aforementioned tack numbers 7, 12, and 1, which came less than 1 cm close to the pericardium, were all adjacent to the critical zone, again pointing out a particular area. Tack numbered 12 belonged to the anterior graft fixation. Although it was 0.79 cm close to the pericardium, tack number 12 was the only nonoffensive tack among seven tacks applied for the fixation of the anterior graft. Surgically, it was placed about 1 cm posteroinferior to the offending “anterior tack line.” Tack numbers 7 and 1 were the most proximally placed tacks at both sides of the arms of the U-shaped graft (Supplementary Video S5). They were in fact applied about 1 cm posteroinferior to the offending “anterior tack line” at both sides of the hiatal opening. This had surgical importance as all other seven tacks used for the fixation of U-shaped posterior graft were consistently inferior, again following a pattern. All the nearest offenders actually represented a transitional area, which was in continuity between the offensive “critical” and “safe” zones, where tacks became inferior and irrelevant.

The shortest measurable distance between tack number 3 and the vena cava was 1.2 mm. The shortest distance between the tacks numbered 4, 5, and 6 and aorta was 0.63, 0.65, and 0.39 cm, respectively (Supplementary Video S4).

## Discussion

CT resulting from perihiatal abdominal surgery represents a particularly challenging subset of traumatic CTs with extremely high mortality rates. Small case studies reported mortality rates between 37.5% and 66.6%.^4,5,8^ Actual mortality rate can even be higher as some deaths go unreported in peer-reviewed journals, which was shown by Frantzides and Welle^8^ by cross-referencing the FDAs Manufacturer and User Facility Device Experience (MAUDE) data with the published reports. Differing from the vast majority of acute CTs wherein there is a chest trauma or another usual suspect, an unrecognized injury occurring during abdominal surgery under mechanical ventilation further complicates the management.

The risk involved with tacking, stapling, or suturing the diaphragm is known because its thickness ranges between 1.5 and 5.4 mm and averages only 2.9–3 mm at the tendinous portion.^15^ Nevertheless, 15 mmHg pneumoperitoneum and reverse Trendelenburg positioning of the patient affect anatomy. A stretched diaphragm will be thinner and should be closer to the pericardium. The helical tack has had a clear warning note attached since 2004, discouraging its use on surfaces with <4 mm in thickness.^16^ Recently, additional warnings contraindicated their use on diaphragm in vicinity to the pericardium.^17^

**FIG. 3.**
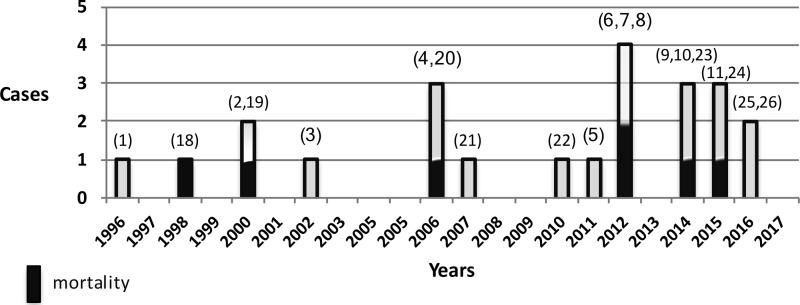
Annual distribution of published cases and mortalities from iatrogenic cardiac tamponade as a result of perihiatal surgery. Numbers in parentheses represent references.

In contrast, a detailed PubMed search identified LARS and perihiatal diaphragmatic graft fixation as the leading causes of CT among all 23 reported cases occurring during upper abdominal surgery.^1–11,18–26^ Fixation of a graft to reinforce a hernia repair (hiatal,^2–9,11^ diaphragmatic,^21,23,26^ or incisional^8,22,24^) was the primary reason of injury in 17 of the 23 reported cases. The literature also indicates helical tacks as the most common predators among all fixing devices as they were responsible from 13 of the reported tamponades,^3–9,21–23,25,26^ resulting in mortality in 4.^4,8,23^ Ironically, despite the mentioned warnings about the use of fixators near the diaphragm, neither the number of reported incidents nor the mortality rate seems to change^1–11,18–26^ (Fig. 3).

In addition to CT, the unsolved problem of mesh erosion, which debilitates a number of patients on the long run, is also a serious issue.^27^ Although not a single mesh erosion became clinically evident or diagnosed during our entire series, we are unable to say that we have a 0% rate as an erosion may remain asymptomatic and, therefore, undiagnosed unless specifically searched for which was beyond the scope of this article.

It is, therefore, reasonable to assume that the temptation to avoid “hernia” recurrence is the reason why surgeons continued to overlook the possible risks of fixing a graft to the diaphragm. This temptation is actually evidence based. Mesh augmentation is shown to decrease the recurrence rate when dealing with large hiatal hernias in all four randomized controlled trials.^28–31^

Upon acting as the medical expert in two iatrogenic CTs due to a fixator injury, which were diagnosed at autopsy, Frantzides suggested that straight hernia staplers rather than helical tacks should be used as fixators, and they should be applied “primarily posteriorly.”^8^ To our knowledge, only Frantzides mentioned the dangers of graft fixation anterior to the hiatal opening.

The present radiosurgical study confirmed Frantzides's foresight by providing data on the anatomical vulnerability of the area covered by the anterior rectangular strip graft (Supplementary Video S5). In this “critical zone,” even the shortest 3.8 mm tack application was not only a dangerous radiological consistency but also a life-threatening clinical burden. It was a radiological consistency, since 6 out of 7 tacks used for anterior graft fixation were the sole offenders among all 16 tacks. Clearly, the tamponade would have been prevented with the avoidance of the anterior graft (Supplementary Video S5).

From a surgical point of view, the transition of tacks from being “offensive” to “nonoffensive and irrelevant” is quite abrupt and covers a horizontal area with a width of <1 cm, which can be referred as the “potentially critical zone” (Supplementary Video S5). This is especially true if we take the dynamic mechanics of ongoing laparoscopic surgery into account. Surgical video shows that this occurs around the midesophageal level upon the completion of the wrap and on the horizontal axis. This area actually neighbors the posterior border of the diaphragmatic surface of the heart. Anterior to it, the base of the heart would be directly facing the central tendon of the diaphragm, whereas posteroinferiorly, the heart becomes anatomically irrelevant.

The critical zone, therefore, represents an area, where there is no guarantee for the safe application of a tack, stapler, or a stitch, because as shown radiologically in the presented case, there might be no definable diaphragm in that area. Other fixators, including straight hernia staplers,^2,11^ stitches,^4,10,19^ and even a liver retractor,^1^ were reported to cause CT, all pointing out an anatomical constraint rather than the type of the offender. Skill of the surgeon may have a secondary role, but the thickness of the diaphragm will be the primary determinant of the outcome. Since our reported complication occurred during the surgeon's 1265th case, technical experience was surely not enough to avoid the injury once a tack was fired in the critical zone.

Therefore, usage of any graft fixator within the critical zone must be abandoned. Furthermore, extreme caution is warranted even when there is an absolute need for stitching. Given the dynamics of real-time surgery, the nearest threat comes from the “potentially critical zone” and this zone should also be avoided. Supplementary Video S5 shows the “safe zone,” where we can use graft fixators for the application of a posterior graft, provided that the most cephalad tacks at both arms of the U-shaped graft are not applied. A single graft with a concentric hole can also be applied circumferentially, without any fixation in the critical zones. Doing so will efficiently cover the anterior defect without any need for anterior fixation.

The “safe zone” definition is certainly arguable because a tack was only 1.2 mm from the vena cava, and others about 0.5 cm from the aorta. To our knowledge, no previous fixator-related great vessel injury has been reported so far, but obviously, great caution is warranted.

LARS, the principal cause of CT resulting from abdominal surgery,^1–11^ is a common procedure worldwide as it is the only chance for cure in patients with erosive GERD. LARS, for its most part, is performed to allow symptomatic relief for a benign disorder, and, therefore, should never result in mortality as far as the risk/benefit ratio is concerned. In particular, any specific instrumental risk is unacceptable and must be prevented.

Although it is known that the definition and reporting of a complication are easy at its extremes and become controversial in case of a minor problem, Frantzides and Welle^8^ excellently showed that even deaths related to graft fixation go unreported in the peer-reviewed medical journals. The unreported deaths will remain a problem, but also interestingly no previous report has provided information about the incidence of cardiac injury during LARS. This study revealed an overall incidence of 0.076% in 1302 consecutive LARS procedures. In mesh-augmented cases however, the incidence was 0.10%. It is noteworthy that no tamponade occurs when either no grafting or only posterior grafting was implemented. In 43 anteriorly grafted patients however, the incidence was 2.3%. The rest 42 patients who had an anteriorly tacked second graft were only lucky as they probably had a thicker diaphragm.

Majority of the graft fixation-related CTs to date have resulted from a helical tack.^3–9,21–23,26^ The Society of American Gastrointestinal and Endoscopic Surgeons' report identified tacks as the second choice for graft fixation, following suturing, which were preferred by 23.9% and 56.4% of the participants, respectively.^32^ Strikingly, helical tacks are causing more deadly problems than more commonly used suturing. The first report that specifically warned about the danger of a helical tack during LARS was published in 2002.^3^ As stated, ironically, numerous reports have repeated the same warning until now.^3–9,21,23,25,26^

Helical tacks are applied under manual pressure against the tissue, causing further thinning down, and their deployment happens in an “all or none” manner with very effective drilling capacity that should never be underestimated. In the critical zone, where a pounding heart applies counter pressure during a systole, “helical tacks” are extremely risky and their usage must be abandoned.

In conclusion, CT during LARS is fortunately rare and there is reason to believe that it is avoidable provided that the specific anatomical constraint is totally addressed. Findings of the anatomical study suggest that avoidance of graft fixation anterior to the hiatal opening during LARS will certainly decrease, if not eliminate, the risk of highly mortal CT.

## Supplementary Material

Supplemental data

Supplemental data

Supplemental data

Supplemental data

Supplemental data
