# Supplementary material for: Cardiac Tamponade as a Life-Threatening Complication of Laparoscopic Antireflux Surgery: The Real Incidence and 3D Anatomy of a Heart Injury by Helical Tacks
Source: J Laparoendosc Adv Surg Tech A. 2018 Sep 11;28(9):1041–6. doi: 10.1089/lap.2017.0713 (PMC6157358; doi:10.1089/lap.2017.0713)
Supplement: Supplemental data [file Supp_video3.zip › Supp_video3.pdf]

SUPPLEMENTARY VIDEO S3. Axial, sagittal, and coronal tomography sections and targeted marking.
